# Genome Sequence of *Marichromatium gracile* YL-28, a Purple Sulfur Bacterium with Bioremediation Potential

**DOI:** 10.1128/genomeA.00288-16

**Published:** 2016-05-05

**Authors:** Xiaobo Zhang, Chungui Zhao, Xuan Hong, Shicheng Chen, Suping Yang

**Affiliations:** a Department of Bioengineering and Biotechnology, Huaqiao University, Xiamen, China; b Department of Microbiology and Molecular Genetics, Michigan State University, East Lansing, Michigan, USA

## Abstract

The draft genome sequence of *Marichromatium gracile* YL-28 contains 3,840,251 bp, with a G+C content of 68.84%. The annotated genome sequence provides the genetic basis for revealing its role as a purple sulfur bacterium in the harvesting of energy and the development of bioremediation applications.

## GENOME ANNOUNCEMENT

*Marichromatium gracile* YL-28 was isolated from an intertidal sediment (collected from an inshore mangrove) in Fujian, China (1). The genus *Marichromatium* belongs to the family *Chromatiaceae*, which was proposed by Imhoff et al. (2). Currently, the genus contains only five species. *M. gracile* YL-28 is the first isolate collected from an inshore mangrove. *M. gracile* YL-28 produced a large amount of rhodopins as dominant carotenoids when anaerobically grown in the light (1). *M. gracile* YL-28 was capable of using reduced sulfur compounds, nitrogen compounds, or molecular hydrogen as electron donors for growth, which was consistent with the native environments in inshore mangrove systems (1). The draft genome sequence will provide further insight into the physiology and metabolic potentials in *M. gracile* YL-28 and related species.

The draft genome was sequenced by Illumina HiSeq 2500 paired-end sequencing technology at Majorbio (Shanghai, China). The reads were assembled using SOAP*denovo* (version 2.04) and further edited by using GapCloser (version 1.12). Removal of short contigs and sequences with potential contamination resulted in 120 contigs with a *N*_50_ of 93,809 bp. The maximum contig size was 176,489 bp. The assembled genome comprises 3.8 million nucleotides, with a G+C content of 68.84%. Gene annotation was carried out by the NCBI Prokaryotic Genome Annotation Pipeline (PGAP) (http://www.ncbi.nlm.nih.gov/genome/annotation_prok/). A total of 3,361 genes yielded a coding capacity of 3.3 million nucleotides (genes/genome, 86.4%). There were at least 69 RNA sequences, including 56 tRNAs, 9 rRNAs, and 4 noncoding RNAs (ncRNAs) in the genome.

Many genes encoding photosynthetic complex assembly/reaction center proteins were identified in the draft genome. We also found protein-coding genes related to the sulfur oxidation/reduction pathways, such as sulfur oxidation *c*-type cytochrome genes (*soxA* and *soxX*) and sulfur reduction protein genes (*dsrE* and *dsrE*). *hemA* and *hemL* genes encoding glutamyl-tRNA synthetase (Gltx) and glutamyl-tRNA reductase (Gtr) were annotated in the draft sequence, indicating that *M. gracile* YL-28 was possibly able to synthesize 5-aminolevulinic (ALA) through the C_5_ pathway but not the Shemin pathway (3). Nitrite reductase and nitric oxide reductase genes also exist in the genome of *M. gracile* YL-28.

### Nucleotide sequence accession numbers.

The genome sequence of *M. gracile* YL-28 was deposited in the GenBank database under the accession no. LSYU00000000. The BioProject designation for this project is PRJNA312715. The BioSample accession no. is SAMN04505658.

## References

[1] Zhao J, Fu Y, Zhao C, Yang S, Qu Y, Jiao N 2011 Identification and properties of a purple sulfur bacterium with rhodopin as predominant carotenoid from mangrove. Acta Microbiol Sin 51:1318–1325. 22233052

[2] Imhoff JF, Suling J, Petri R 1998 Phylogenetic relationships among the *Chromatiaceae*, their taxonomic reclassification and description of the new genera *Allochromatium*, *Halochromatium*, *Isochromatium*, *Marichromatium*, *Thiococcus*, *Thiohalocapsa* and *Thermochromatium*. Int J Syst Evol Microbiol 48:1129–1143. 10.1099/00207713-48-4-1129 9828415

[3] Willows RD, Kriegel AM Biosynthesis of bacteriochlorophylls in purple bacteria, p 57–79. *In* Hunter CN, Daldal F, Thurnauer MC, Beatty JT (ed), The purple phototrophic bacteria. Springer Netherlands, Dordrecht, The Netherlands.

